# Use of multislice computed tomography in the diagnosis of annular constrictive pericarditis

**DOI:** 10.1590/0100-3984.2015.0151

**Published:** 2016

**Authors:** Bruno Hochhegger, Klaus L. Irion, Gláucia Zanetti, Edson Marchiori

**Affiliations:** 1 Universidade Federal de Ciências da Saúde de Porto Alegre (UFCSPA), Porto Alegre, RS, Brazil; 2 Liverpool Heart and Chest Hospital - NHS Trust, Liverpool, United Kingdom; 3 Universidade Federal do Rio de Janeiro (UFRJ), Rio de Janeiro, RJ, Brazil

**Figure 1:**
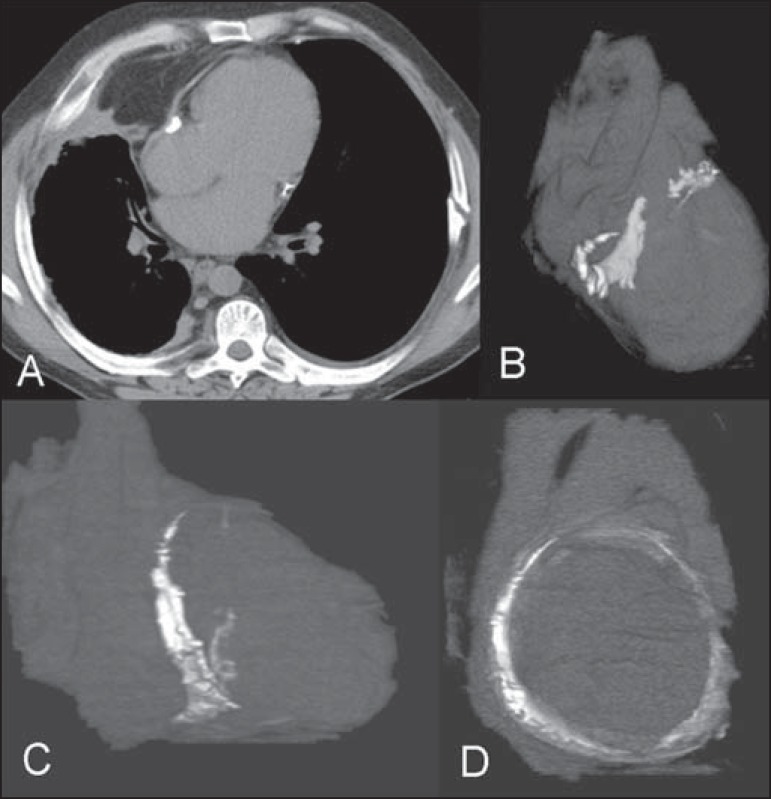
Axial computed tomography image (**A**) and volume-rendered reconstructions (**B-D**) showing a calcified pericardial band encircling the left ventricular cavity at the level of the atrioventricular groove.

*Dear Editor*,

A 65-year-old man with a history of pleural tuberculosis was referred to our outpatient clinic due to respiratory difficulty. He presented with worsening dyspnea on minimal exertion. Examination confirmed that the patient was experiencing mild respiratory difficulty; his respiration rate was 25 breaths per minute, and his heart rate was 98 beats per minute. Cyanosis, jaundice, and signs of heart failure were absent, and other systems appeared normal. Multislice computed tomography showed a calcified pericardial band encircling the left ventricular cavity at the level of the atrioventricular groove (Figure 1).

Complete pericardiectomy was performed successfully and the postoperative course was uneventful. Histopathologic examination of the excised pericardium showed fibrocollagenous thickening with areas of hemorrhage and heavy calcific deposits. No areas of granuloma or vasculitis were identified. The final diagnosis was annular constrictive pericarditis.

Constrictive pericarditis is characterized by thick pericardial fibrosis and frequent calcification that progressively impairs diastolic filling of the heart, with associated symptoms of heart failure^(1)^. Annular constrictive pericarditis is extremely rare, and few similar cases have been reported^(1)^. Previous pericardiectomy, congenital heart disease, and complications of tuberculosis may be the leading causes of this condition. Depending on the location of the pericardial constriction, the clinical presentation of localized constriction may differ, including obstruction of the right ventricular outflow tract, coronary obstruction, and pulmonary stenosis^(1-3)^. The imaging evaluation of cardiovascular calcifications has been the subject of a series of recent publications in the Brazilian radiology literature^(4-7)^. Multislice computed tomography may be an important tool for the precise identification of annular constrictive pericarditis^(8)^.
